# Polyethylenimine Increases Antibacterial Efficiency of Chlorophyllin

**DOI:** 10.3390/antibiotics11101371

**Published:** 2022-10-07

**Authors:** Faheem Ahmad Akif, Mona Mahmoud, Binod Prasad, Peter Richter, Azizullah Azizullah, Muhammad Qasim, Muhammad Anees, Marcus Krüger, Susanne Gastiger, Andreas Burkovski, Sebastian M. Strauch, Michael Lebert

**Affiliations:** 1 Department of Microbiology, Kohat University of Science and Technology (KUST), Kohat 26000, Pakistan; 2 Gravitational Biology Group, Department of Biology, Cell Biology Division, Friedrich-Alexander-Universität, Erlangen-Nürnberg, 91058 Erlangen, Germany; 3 Dairy Department (Microbiology Lab.), National Research Centre, Cairo 12622, Egypt; 4 Department of Biology, Microbiology Division, Friedrich-Alexander-Universität, Erlangen-Nürnberg, 91058 Erlangen, Germany; 5 Department of Botany, Kohat University of Science and Technology (KUST), Kohat 26000, Pakistan; 6 Environmental Cell Biology Group, Department of Microgravity and Translational Regenerative Medicine, Otto-von-Guericke University, 39106 Magdeburg, Germany; 7 Postgraduate Program in Health and Environment, University of Joinville Region, Joinville 89219-710, SC, Brazil; 8 Space Biology Unlimited S.A.S., 33000 Bordeaux, France

**Keywords:** chlorophyllin, polyethlyenimine, synergistic effect, photodynamic antimicrobial activity

## Abstract

Polyethylenimines (PEIs), a group of polycationic molecules, are known to impair the outer membrane of Gram-negative bacteria and exhibit antimicrobial activity. The outer membrane of Gram-negative strains hinders the uptake of photosensitizer chlorophyllin. In this study, we report chlorophyllin and branched PEI combinations’ activity against *Escherichia coli* strains DH5α and RB791, *Salmonella enterica* sv. Typhimurium LT2, and *Bacillus subtilis* 168. The minimal bactericidal concentration (MBC) was determined by plating cells treated with different concentrations of PEI and chlorophyllin on agar and monitoring their growth after 24 h. All tested combinations of PEI and chlorophyllin were lethal for *S. enterica* after 240 min of incubation in light, whereas PEI alone (<100 µg mL^−1^) was ineffective. In the darkness, complete inhibition was noted with a combination of ≥2.5 µg mL^−1^ chlorophyllin and 50 µg mL^−1^ PEI. If applied alone, PEI alone of ≥800 µg mL^−1^ of PEI was required to completely inactivate *E. coli* DH5α cells in light, whereas with ≥5 µg mL^−1^ chlorophyllin, only ≥100 µg mL^−1^ PEI was needed. No effect was detected in darkness with PEI alone. However, 1600 µg mL^−1^ PEI in combination with 2.5 µg mL^−1^ resulted in complete inactivation after 4 h dark incubation. PEI alone did not inhibit *E. coli* strain RB791, while cells were inactivated when treated with 10 µg mL^−1^ chlorophyllin in combination with ≥100 µg mL^−1^ (in light) or ≥800 µg mL^−1^ PEI (in darkness). Under illumination, *B. subtilis* was inactivated at all tested concentrations. In the darkness, 1 µg mL^−1^ chlorophyllin and 12.5 µg mL^−1^ PEI were lethal for *B. subtilis*. Overall, PEI can be used as an antimicrobial agent or potentiating agent for ameliorating the antimicrobial activity of chlorophyllin.

## 1. Introduction

The discovery of antibiotics in the last century and their subsequent application against pathogens resulted in a significant reduction in infectious diseases and an increase in the average life expectancy [1]. However, the overuse and misuse led to the development of resistance against almost all available antibiotics [2]. Today, antibiotic resistance is a significant threat to public health, and if no countermeasures are taken, it may cause millions of fatalities in the following decades [3]. New approaches must be developed to mitigate antibiotic resistance and infectious bacterial diseases [2,4,5,6].

Photodynamic inactivation of bacteria, also known as antimicrobial photodynamic therapy (Apdt) or photodynamic antibacterial chemotherapy (PACT), is gaining interest in mitigating bacterial pathogens [7,8,9,10]. Here, a light-sensitive substance called photosensitizer (PS) is applied. Upon illumination with an appropriate wavelength of light, the PS is excited to a singlet or triplet state. In the presence of oxygen, the triplet state energy may transfer in two types of reactions. In type I reactions, the transfer of an activated electron leads to the formation of reactive oxygen species (ROS.), such as hydrogen peroxide (H_2_O_2_) or hydroxyl radicals (•OH). The energy forms singlet oxygen (1O2) in type II reactions, a highly reactive ROS [11,12]. These reactive molecules are directly or indirectly involved in the photodynamic activity against microorganisms. For example, cationic photodynamic agent ZnPc(T.A.P.)_4_ showed potent activity against Gram-negative bacteria with negligible effects on mammalian cell lines [13]. Chen et al. (2020) developed membrane-anchoring photosensitizers with aggregate-induced emission, displaying strong singlet oxygen production in the aggregate form [14]. By anchoring, these particles adhere to the bacterial cell membrane and show 99.8% higher killing efficiency in *Escherichia coli* and *Staphylococcus aureus* [14]. Promising natural photosensitizers are chlorophylls and their derivatives. Chlorophyllin, a water-soluble derivate of chlorophyll, inactivates mosquito larvae and water-born parasites, such as the fish parasite *Ichthyophthirius multifiliis* or *Ergasilus* sp. [15,16,17]. In addition, chlorophyll and its derivatives (especially water-soluble chlorophyllin) exhibit pronounced photodynamic properties against bacteria [9,18,19,20].

Gram-negative strains are less susceptible to photodynamic treatment with chlorophyllin than planktonic Gram-positive bacteria, such as *Bacillus subtilis* [9]. This difference in sensitivity to chlorophyllin is perhaps due to the presence of the second cell membrane in Gram-negative bacteria [9,10]. The outer membrane-deficient *E. coli* strains (NR698) exhibit higher sensitivity to chlorophyllin than Gram-positive bacteria [9], indicating that the outer membrane acts as a barrier to the uptake of chlorophyllin. Furthermore, the sensitivity of Gram-negative bacteria to chlorophyllin is higher in the presence of a cell wall destabilizing antibiotic, such as colistin, compared to only chlorophyllin [10]. Colistin is a last-resort antibiotic with potential side effects on human health [21,22]. As a polycationic molecule, colistin interacts with the negatively charged lipopolysaccharide of the outer membrane leading to membrane destabilization [23] and the uptake of chlorophyllin into cytoplasm and growth inhibition of bacterial cells [9,10].

Similarly to colistin, polyethylenimines (PEIs) are a group of polycationic molecules. PEIs have branched or linear configurations due to numerous amino groups [24]. PEIs exhibit antimicrobial properties [25,26]. Similarly to colistin, PEI impairs the outer membrane of Gram-negative bacteria by interaction with lipopolysaccharides [27,28]. Initial experiments showed that branched PEI considerably increases the photodynamic activity of chlorophyllin. In this study, we report the antimicrobial effect of different combinations of PEI and chlorophyllin against Gram-positive and Gram-negative bacterial strains.

## 2. Results

### 2.1. Effect of PEI and Chlorophyllin on *Salmonella enterica* serovar Typhimurium

**Figure 1:**
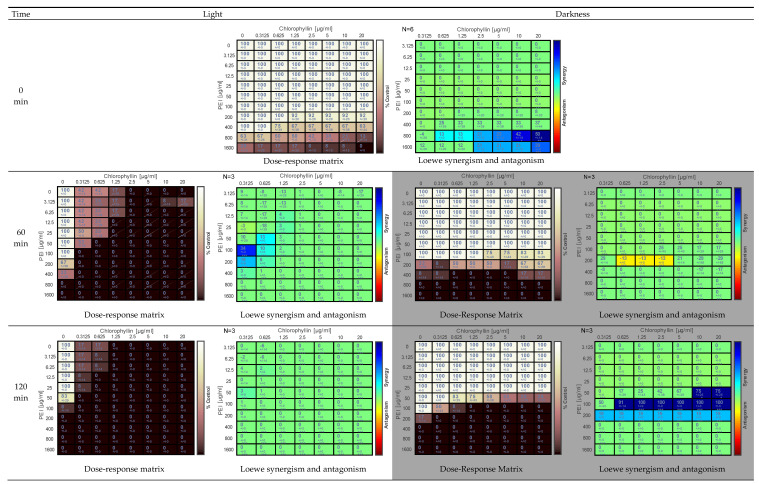

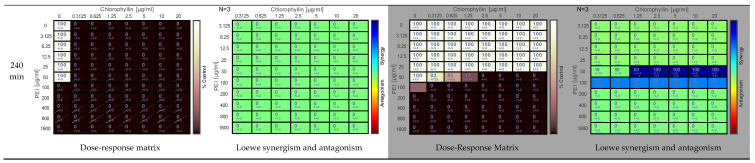
Effect of PEI and chlorophyllin combinations on *Salmonella enterica* serovar Typhimurium upon illumination with a blue-red light source (12.7 mW cm^−2^) and in darkness at 0 min, 60 min, 120 min, and 240 min of incubation. The dose-response matrix indicates the percentage of eradication (the darker the color, the higher the inhibitory effect). The Loewe synergism and antagonism for PEI-chlorophyllin combinations against *S. enterica* serovar Typhimurium were determined using the Combenefit software. N represents the number of biological replicates in the matrix. Asterisks represent statistical significance: *: *p* < 0.05, **: *p* < 0.01 and ***: *p* < 0.0001.

The combination of PEI and chlorophyllin eradicated *S. enterica* sv. Typhimurium considerably fast in light (12.7 mW m^−2^ blue-red) and dark incubation. In light incubation, the inhibitory effect was observed at the 0 min analysis (corresponding to 15 min considering the time from sample preparation to the first sample point) at concentrations of 200–1600 µg mL^−1^ and 1.25–20.0 µg mL^−1^ of PEI and chlorophyllin, respectively (Figure 1). After 60 min of incubation, higher concentrations of PEI alone (≥800 µg mL^−1^) eradicated the cells. After 240 min of incubation, all combinations of chlorophyllin and PEI were lethal for the cells, and colonies could only be recovered from cells treated with PEI alone at concentrations <100 µg mL^−1^. Similarly, in dark incubation, chlorophyllin/PEI combinations were highly effective against this strain (Figure 1). After 240 min of incubation, complete cell inactivation was observed at ≥200 µg mL^−1^ PEI concentrations. The addition of chlorophyllin increased the antibacterial effect. At chlorophyllin concentrations ≥2.5 µg mL^−1^, complete inactivation of bacteria was achieved in combination with 50 µg mL^−1^ PEI. Combenefit analysis revealed synergism in some combinations of PEI-chlorophyllin in light and dark incubation conditions (Figure 1).

### 2.2. Effects of PEI and Chlorophyllin on *Escherichia coli* DH5α

**Figure 2:**
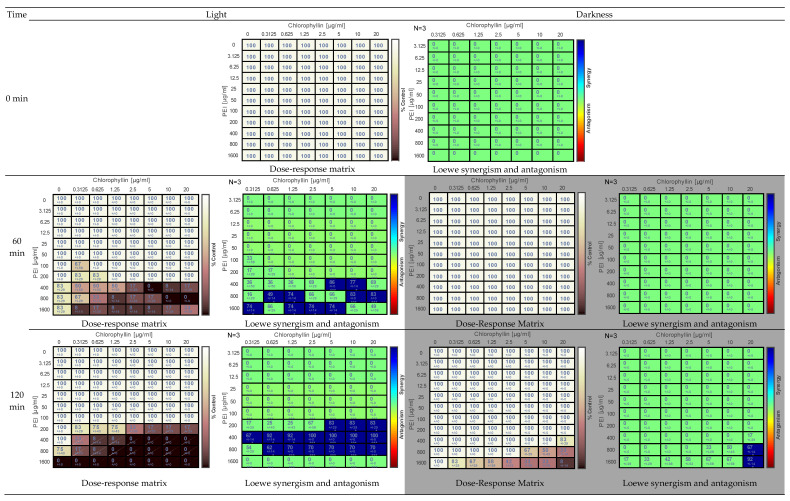

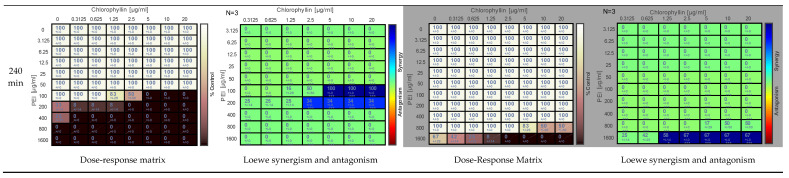
Effect of PEI and chlorophyllin combinations on *E. coli* DH5α upon illumination with a blue-red light source (12.7 mW cm^−2^) and in darkness at 0 min, 60 min, 120 min, and 240 min. of incubation. The dose-response matrix indicates the percentage of eradication (the darker the color, the higher the inhibitory effect). The Loewe synergism and antagonism for PEI-chlorophyllin combinations against *E. coli* DH5α were determined using the Combenefit software. N represents the number of biological replicates in the matrix. Asterisks represent statistical significance: *: *p* < 0.05 and ***: *p* < 0.0001.

Similar to *S. enterica* sv. Typhimurium inactivation of *E. coli* DH5α was achieved by the combination of PEI-chlorophyllin both in light and darkness (Figure 2). However, this strain was less sensitive to PEI-chlorophyllin concentrations. After 240 min, under illumination, cells were inactivated entirely at concentrations ≥800 µg mL^−1^ of PEI alone compared to ≥100 µg mL^−1^ in the case of *S. enterica* (Figure 2). Complete inactivation of *E. coli* DH5α was observed at ≥100 µg mL^−1^ and ≥5 µg mL^−1^ of PEI and chlorophyllin concentrations, respectively (Figure 2). No eradication effect was noted in any of the tested concentrations of chlorophyllin. In dark incubation, complete inactivation was observed only at combinations of 1600 µg mL^−1^ PEI and ≥ 2.5 µg mL^−1^ (Figure 2). Combenefit analysis revealed synergism at combinations of lower concentrations of chlorophyllin and PEI (Figure 2).

### 2.3. Effect of PEI and Chlorophyllin on *Escherichia coli* RB791

Based on preliminary experiments, 10 µg mL^−1^ chlorophyllin concentration was used in combination with different concentrations of PEI to determine the sensitivity of *E. coli* strain RB791 to chlorophyllin and PEI. Cells incubated in dark conditions were treated with higher concentrations of PEI (0–800 µg mL^−1^) than cells in light (0–100 µg mL^−1^). PEI alone did not exhibit any lethal effect on the strain RB791, even after incubation for 240 min in light and dark conditions. However, in combination with 10 µg mL^−1^ chlorophyllin, apparent eradication was observed under light and dark incubation conditions (Table 1). Already, after 60 min, growth inhibition was detected and after 180 min, complete eradication was observed at 100 µg mL^−1^ and 800 µg mL^−1^ of PEI in light and darkness, respectively (Table 1).

### 2.4. Effect of PEI and Chlorophyllin on *Bacillus subtilis* 168

**Figure 3:**
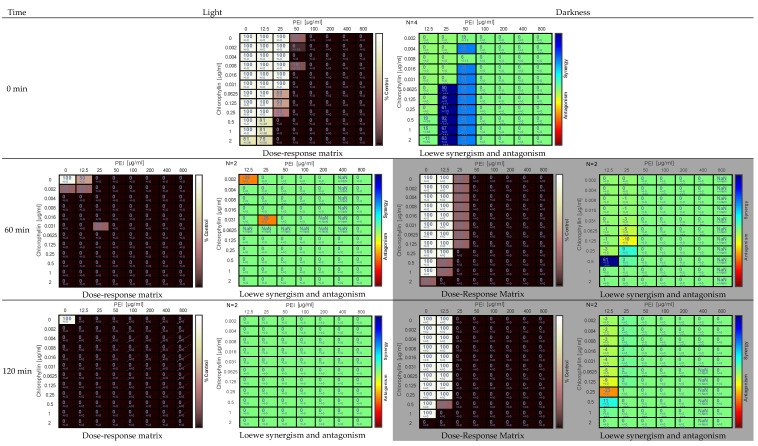
Effect of PEI and chlorophyllin combinations on *Bacillus subtilis* 168 upon illumination with a blue-red light source (12.7 mW cm^−2^) and in darkness at 0 min, 60 min, and 120 min of incubation. The dose-response matrix indicates the percentage of eradication (the darker the color, the higher the inhibitory effect). The Loewe synergism and antagonism for PEI-chlorophyllin combinations against *B. subtilis* 168 were determined using the Combenefit software. N represents the number of biological replicates in the matrix. Asterisks represent statistical significance: ***: *p* < 0.0001.

The Gram-positive model bacterium *B. subtilis* was inactivated considerably quickly in the presence of chlorophyllin and PEI combinations (Figure 3). The sensitivity of *B. subtilis* against PEI and chlorophyllin was much higher in light conditions. Complete inhibition of cells was observed at 0 min of analysis (corresponds to 15 min of sample preparation and sampling in low light conditions) and PEI concentrations of 100 µg mL^−1^ and above in light incubated cells. At 0 min, chlorophyllin alone did not exhibit an immediate effect. However, 60 min sample analysis showed that ≥0.004 µg mL^−1^ of chlorophyllin alone completely eradicated the cells in light conditions (Figure 3). Light incubated cells were already entirely eradicated at the lowest tested concentrations of chlorophyllin (0.002 µg mL^−1^) and PEI (>12.5 µg mL^−1^). The cells were killed within 120 min of incubation in light at all the tested concentrations of PEI (12.5–800 µg mL^−1^) and chlorophyllin (0.002–2.000 µg mL^−1^) combinations. Only control cells (no chlorophyllin and PEI) showed growth. Pronounced synergistic effects were detected above all at 0 min. Due to the strong effect of both components on bacteria survival, Combenefit analysis can hardly detect synergism at later time points. Complete (100%) inhibition of cells was observed at 2 µg mL^−1^ of chlorophyllin alone and >12.5 µg mL^−1^ of PEI alone at 120 min of incubation in darkness (Figure 3).

### 2.5. Determination of the Effect of PEI and Chlorophyllin Related to Cell Density

All the above experiments were performed with a defined culture density (OD_600nm_) of 0.01. The MBC of different concentrations of PEI (1.95–250 µg mL^−1^) + chlorophyllin (0–30 µg mL^−1^) was investigated against different initial cell densities (OD_600nm_ = 0.0003–0.73). For this study, freshly grown *E. coli* DH5α was employed, and the experiment was performed in 96-well microtiter plates. In light, higher concentrations of PEI-chlorophyllin showed stronger eradication effects on higher cell concentrations. Cell densities ≥ 0.0007 (OD_600nm_) incubated in light conditions were completely eradicated at the highest tested concentration of PEI (250 µg mL^−1^) (Table 2). Chlorophyllin alone was not effective in both light and dark incubation conditions. However, in the presence of PEI (≥125 µg mL^−1^) and under illumination, a strong effect was noticed at 30 µg mL^−1^ chlorophyllin for the tested highest cell density (i.e., OD_600nm_ = 0.73). Dark controls, in contrast, were hardly affected at the applied concentration range.

## 3. Discussion

Destabilizing the outer membrane of Gram-negative bacteria with substances such as lactic acid, EDTA, and PEI is a promising tool against pathogenic bacteria, because the outer membrane is an effective barrier against xenobiotics and other harmful substances [10,29,30]. This study demonstrates the inhibitory effects of the membrane destabilizer PEI and the photosensitizer chlorophyllin alone and in combinations against Gram-positive and Gram-negative bacterial strains. At higher concentrations, PEI alone was found to inactivate Gram-negative *E. coli* DH5α and *S. enterica*, as well as Gram-positive *B. subtilis.* However, combinations of PEI and chlorophyllin exhibited considerably stronger effects than either of these compounds. Similarly to colistin, PEI interacts with and destabilizes the outer membrane lipopolysaccharides of Gram-negative bacteria [10,27,28]. Chlorophyllin enters the cell through the destabilized outer membrane, and upon exposure to light, photodynamic destruction of cells occurs [10,18,27,28,31]. Porphyrins, such as chlorophylls, are potent photodynamic molecules [18,31]. Cellamare et al. (2013) reported that photoactivation of a chlorophyll/cyclodextrin complex yields the formation of superoxide and hydroxyl radicals (originating from type I photodynamic reactions) as well as singlet oxygen to a lesser extent (from type II photodynamic reactions) [32]. The lifetime of superoxide, singlet oxygen, and hydroxyl radicals are ~1.25 s [33], ~50–700 µs [34], and ~10−10 s, respectively [33]. During their lifetime, reactive oxygen species can cover distances of approximately 50 µm (superoxide), 1.2 µm (singlet oxygen), or 0.4 nm (hydroxyl radicals), respectively, before returning to the ground state (according to the formula $x\;\approx \;\sqrt{t\times 2D}$, where *x* is the distance, *t* is the lifetime, and *D* is the diffusion coefficient of 1 × 10^−5^ cm^2^ [35]). Consequently, only superoxides (e.g., H_2_O_2_) can affect cellular targets at a longer distance. This led to the assumption that chlorophyllin present outside the cell is less effective in producing cell damage than chlorophyllin accumulated in the cytoplasm. The outer membrane of Gram-negative bacteria was found to be a barrier for chlorophyllin, significantly reducing its efficiency [9,10]. The treatment of Gram-negative bacteria with a combination of chlorophyllin and a cell wall destabilizing agent colistin led to the uptake of chlorophyllin into the cell cytoplasm and increased cytotoxicity, leading to photodynamic cell death.

Our data show that branched PEI has significant potential as synergistic or potentiating agents against bacteria. However, in the course of experiments using the cell wall destabilizing agent colistin in combination with chlorophyllin, the necessary concentration (weight per volume) of colistin for bacterial eradication was found to be far below that required by branched PEI. *E. coli* DH5α was eradicated after 4 h of incubation with concentrations of 0.5 µg/mL colistin and 5 µg/mL chlorophyllin compared to 100 µg/mL PEI in combination with the same concentration of chlorophyllin. The difference is probably due to the different structures of the molecules. Colistin is smaller (1155.4 g/mol) compared to the branched PEI (about 25 kDa) employed in this study. In addition, the colistin molecule contains hydrophilic as well as lipophilic components, while the polybasic amine polymer branched PEI (B-PEI) is water soluble and lacks lipophilic components [10,36]. PEI is available in various forms (linear, branched, and dendrimer) and sizes (800–270,000 kDa, Sigma-Aldrich, S. Louis, MO, U.S.A.), which were not yet tested for their eradication potential in combination with chlorophyllin. Additionally, chemical modifications have the potential for increasing the effectivity of PEI. Yin et al. (2017) synthesized various quaternized forms of PEI800 by nucleophilic substitution with different alkyl bromide chains [37]. They tested antibacterial effects against the Gram-positive strain MRSA70069 and the Gram-negative *Pseudomonas aerguinosa* 902, as well as membrane depolarization (using the membrane potential-sensitive dye diSC3(5)). All quaternized PEI800-polymers exhibited increased cytotoxicity against bacteria and induced significant membrane depolarization compared to unmodified PEI800. PEI800 FQ C6, a PEI800 fully quaternized with short alkyl groups, was the most effective PEI polymer. In another study, Roest et al. (2015) examined the charging properties of hyperbranched polyurea-polyethyleneimine coatings [25]. After varying the degree of alkylation, the authors concluded that cationic charges alone are ineffective in contact-killing bacteria and needs to be carried by an alkylated nitrogen species. To investigate the effect of PEI on bacteria causing healthcare-associated infections, Azevedo et al. (2014) studied the effect of PEI and PEI-based nanoparticles (synthesized by reductive amination) on various bacterial species (*Staphylococcus aureus*, *Staphylococcus epidermidis*, *Acinetobacter baumannii*) and the yeast *Candida albicans* as well as their ability to form biofilms in a polyurethane catheter-model system [38]. PEI, in particular, inhibited growth and biofilm formation very efficiently, while nanoparticles were found to be less effective in these experiments. In contrast, another study using quaternary ammonium polyethyleneimine- (QA-PEI) nanoparticles resulted in the effective inactivation of *S. aureus* and *E. coli* [39]. The addition of PEI-nanoparticles into resin composite materials showed long-lasting antibacterial and anti-biofilm effects, making PEI interesting for dental prosthetics [40]. Following promising ex vivo experiments, application in the antimicrobial treatment of dental root canals [41] and bone cement for joint replacement surgery was suggested [40].

The Gram-positive model bacterium *B. subtilis* was severely affected by combinations of chlorophyllin and PEI. Under illumination, a complete loss of viability was found after 60 min at all tested concentrations of PEI, chlorophyllin, or their combinations, respectively. Interestingly, the highest tested concentration of chlorophyllin (2 µg/mL) alone did not completely eradicate the cells in darkness; however, combination with 12.5 µg/mL PEI led to complete eradication of the cells. This result comes in agreement with Foxley et al., (2016), who found that susceptibility of methicillin-resistant *S. aureus* (MRSA) strains against ampicillin and vancomycin can be restored in the presence of branched PEI because of its interaction with the wall teichoic acids (WTA) without entering the cells [42]. Generally, Gram-positive bacteria synthesize their cell wall with certain transpeptidases (PBPs, penicillin-binding proteins), which are the target of β-lactams. MRSA, however, possesses a β-lactam-PBP2a, providing resistance against this group of antibiotics. Positively charged amino groups of the branched PEI interact with negatively charged phosphate bridges of WTAs and impede the appropriate orientation of PBP2a, a prerequisite of cell wall synthesis that requires WTAs for its proper activity, thus, implying that the activity of PEI is most likely due to electrostatic interaction [43].

From the above-mentioned data, we can conclude that PEI can be used as an antimicrobial agent or potentiating agent for ameliorating the antimicrobial activity of chlorophyllin or other antibiotics.

## 4. Materials and Methods

### 4.1. Bacterial Strains and Growth Conditions

Experiments were performed with bacterial strains *E. coli* DH5α, *E. coli* RB791, *S.* Typhimurium LT2, and *B. subtilis* 168. Cells were grown overnight (16 ± 2 h) in Luria Bertani (LB) medium at 37 °C. Cell concentration was determined by measuring the culture’s optical density (OD) at 600 nm, and the initial cell concentration was adjusted to OD_600nm_ 0.01 for antimicrobial assay.

### 4.2. Preparation of Chlorophyllin

Chlorophyllin was prepared from spinach, as described previously [9]. In brief, 500 g of frozen spinach was thawed, mixed with 5 g CaCO_3_ to avoid phaeophytin formation, and pressed through a cotton tissue to remove water. In the darkness, the leaves were extracted in 500 mL methanol for 12 h at 4 °C. After filtration, 300 mL petroleum benzene was added, forming a hydrophobic phase on top of the methanolic phase. Subsequent addition of small aliquots of saline water (5% NaCl) forced the chlorophyll into the benzene layer. After the complete transfer of chlorophyll into the benzene phase, phases were separated using a separating funnel. A defined small sample volume was withdrawn, the benzene was evaporated, and the residual chlorophyll was dissolved in a specified volume of methanol. The chlorophyll concentration in the benzene phase was determined according to Lichtenthaler and Wellburn (1983) [44]. Finally, 250 mL benzene-chlorophyll was extracted using 15 mL of 100 mM methanol-sodium hydroxide solution. This treatment cleaves the phytol tail of chlorophyll and transforms it into water-soluble chlorophyllin, which moves into the methanol-sodium hydroxide phase. The volume of the resulting chlorophyllin in the methanol-sodium hydroxide phase was determined, and its concentration was calculated accordingly. All incubations were carried out in the darkness, with sample handling under low light conditions.

### 4.3. Determination of Minimum Bactericidal Effects of PEI and Chlorophyllin Combinations

The minimum bactericidal concentration (MBC) of PEI (25 kDa, branched, cat no. #408727, Sigma-Aldrich, St. Louis, MO, U.S.A.) and chlorophyllin combination was determined as described [9]. In brief, cells were incubated in 96-well plates for defined times with different concentrations of PEI and chlorophyllin. The initial cell concentration was adjusted to an optical density (OD_600nm_) of 0.01. For each experiment, two plates were prepared. One plate was exposed to light at an intensity of 12.7 mW cm^−2^, and the other plate was wrapped with aluminum foil, which was used as a dark control. A spectrum of the light source is shown elsewhere [9]. At defined time points of incubation, 3 µL from each sample were plated on LB agar and incubated overnight at 37 °C. Subsequently, colony formation was determined, where no growth was classified as 100% effective, strongly reduced growth as 66%, reduced growth as 33%, and complete growth as 0% inhibitory effects, if not otherwise indicated. The number of replications is indicated in the corresponding figures. The mean values of individual bactericidal values from independent experiments were calculated and displayed.

### 4.4. Statistical Analysis

The data were analyzed by Combenefit software (version 2.021) https://www.cruk.cam.ac.uk/research-groups/jodrell-group/combenefit (accessed on 23 July 2022). The dose-response matrix shows the eradication percentage (the darker the color, the higher the eradication effect). Loewe synergy model was used to determination of the synergism and the antagonism between the tested combinations. In the Loewe synergy and antagonism matrix, N represents the number of biological replicates.

### 4.5. Determination of PEI/Chlorophyllin Effects on Cell Density

Experiments were performed with overnight (16 ± 2 h) grown cultures of *E. coli* DH5α (OD_600nm_ 2.92). Cell density gradients were prepared in 96-well plates in a horizontal direction (left to right of the plate), with two-fold serial dilution of cells in LB medium from one column to the next. Subsequently, two-fold serial dilution of PEI was in the vertical direction (top to bottom of the plate). The last column represents growth control. Defined chlorophyllin concentrations (1.25, 10, and 30 µg/mL) were added separately. After incubation for 24 h under light (12.7 mW cm^−2^) or darkness, 3 µL from each well were plated on LB agar and incubated overnight at 37 °C as described in Section 4.3. PEI-chlorophyllin combinations were classified as having no eradication effect if any colony was observed on the corresponding inoculated spot, while the complete absence of cells was classified as an effective eradication concentration.

## 5. Conclusions

Combinations of chlorophyllin and PEI exhibit strong antibacterial effects, above all under illumination but to a lower extent also in darkness. The results of this study are in context with observations of other research groups and suggest that the potential for synergistic effects of cell wall destabilizing substances (e.g., polyamines) and photosensitizers may exist. The chemical structures can be modified to improve antimicrobial activity further. Furthermore, the determination of antimicrobial properties and cell toxicity of different PEIs or PEI-nanoparticles are of utter interest. At least under light conditions, antimicrobial effects of chlorophyllin are most likely caused by induction of excessive oxidative stress, which makes the formation of resistant strains very unlikely.

## Figures and Tables

**Table 1:** Effect of different concentrations of PEI (0, 25, 50, 75, and 100 µg mL^−1^ under light conditions and (0, 200, 400, 600, 800 100 µg mL^−1^ under dark conditions, respectively) and 10 µg mL^−1^ chlorophyllin on the viability of *E. coli* RB791 upon illumination with a blue-red light source (12.7 mW cm^−2^) and in darkness at 0 min, 60 min, 120 min, 180 min, and 240 min of incubation. No growth was considered as 0%, growth reduction as 33% (strong) and 66% (moderate), and average growth as 100%. The eradication percentages are presented as the average of three independent experiments. The darker the colors, the higher the effect.

10 µg mL^−1^ Chlorophyllin + PEI Concentration [µg mL^−1^]	100	75	50	25	0	10 µg mL^−1^ Chlorophyllin + PEI Concentrations [µg mL^−1^]	800	600	400	200	0
Illumination						Dark Incubation					
0 min	100	100	100	100	100	0 min	100	100	100	100	100
60 min	33	67	100	100	100	60 min	33	100	100	100	100
120 min	17	33	100	100	100	120 min	17	17	50	50	100
180 min	0	100	100	100	100	180 min	0	33	33	100	100
240 min	0	100	100	100	100	240 min	0	50	67	100	100

**Table 2:** Effect of different concentrations of PEI and chlorophyllin on different cell densities of *E. coli* DH5α incubated under a blue-red light source (12.7 mW cm^−2^) and in darkness for 24 h. The maximum cell densities that are entirely eradicated at a given combination of PEI (vertical concentration gradient in µg mL^−1^) and chlorophyllin (horizontal concentration gradient in µg mL^−1^) are presented (n.e.: no effect).

Light	Initial Cell Densities (OD_600_) Inactivated by PEI-Chlorophyllin Concentrations (µg mL^−1^)			
**CHL→ PEI↓**	**0**	**1.25**	**10**	**30**
**1.95**	n.e.	n.e.	n.e.	n.e.
**3.91**	n.e.	n.e.	n.e.	0.0014
**7.81**	n.e.	n.e.	n.e.	0.0057
**15.63**	n.e.	n.e.	0.0004	0.0114
**31.25**	n.e.	n.e.	0.0057	0.0114
**62.50**	n.e.	0.0007	0.0228	0.1825
**125.00**	n.e.	0.0057	0.0913	0.7300
**250.00**	0.0007	0.1813	0.3650	0.7300
**Dark**	**Initial Cell Densities (OD_600_) Inactivated by PEI-Chlorophyllin Concentrations (µg mL^−1^)**			
**CHL→ PEI↓**	**0**	**1.25**	**10**	**30**
**1.95**	n.e.	n.e.	n.e.	n.e.
**3.91**	n.e.	n.e.	n.e.	n.e.
**7.81**	n.e.	n.e.	n.e.	n.e.
**15.63**	n.e.	n.e.	n.e.	n.e.
**31.25**	n.e.	n.e.	n.e.	n.e.
**62.50**	n.e.	n.e.	n.e.	n.e.
**125.00**	n.e.	n.e.	0.0004	0.0004
**250.00**	n.e.	n.e.	0.0014	0.0014

## Data Availability

Complete raw data are available on request.
